# Supplementary material for: Spectral control of nonclassical light pulses using an integrated thin-film lithium niobate modulator
Source: Light Sci Appl. 2022 Nov 17;11:327. doi: 10.1038/s41377-022-01029-7 (PMC9672118; doi:10.1038/s41377-022-01029-7)
Supplement: Supplementary file 1 — Supplementary Information [file 41377_2022_1029_MOESM1_ESM.pdf]

Supplementary information for
Spectral control of nonclassical light pulses using an integrated thin-film lithium niobate modulator

Di Zhu,[1, 2, †, *] Changchen Chen,[3, †] Mengjie Yu,[1, †] Linbo Shao,[1] Yaowen Hu,[1] C. J. Xin,[1] Matthew Yeh,[1] Soumya Ghosh,[1] Lingyan He,[4] Christian Reimer,[4] Neil Sinclair, [1] Franco N. C. Wong,[3] Mian Zhang,[4] Marko Lončar[1,*]

[1] *John A. Paulson School of Engineering and Applied Sciences, Harvard University, Cambridge, MA 02138, USA*
[2] *Institute of Materials Research and Engineering, Agency for Science, Technology and Research (A*STAR), Singapore 138634, Singapore*
[3] *Research Laboratory of Electronics, Massachusetts Institute of Technology, Cambridge, MA 02139, USA*
[4] *HyperLight Corporation, 501 Massachusetts Ave, Cambridge, MA 02139, USA*
[†] *These authors contributed equally*
[*] *zhu_di@imre.a-star.edu.sg; loncar@seas.harvard.edu*

1. Performance comparison

Table S1 compares our results to some representative experiments in the literature on spectral control of quantum light by electrical means (both electro-optic and piezo-acousto-optic). For electro-optic methods, all past demonstrations use bulk LN modulators, and our work is the only one utilizing integrated TFLN modulator. Our results have the largest frequency shift and highest power efficiency.

Table S1. Performance benchmarking on electrically driven quantum spectral control.

Reference	Methods	Source	Wave-length (nm)	Freq. shift (GHz)	Band-width comp. factor	Freq. shift efficiency	Time lens RF power and frequency
Wright (2017) [1]	EO spectral shearing with bulk LN modulator	Pulsed SPDC	832	± 200	-	141 GHz $W^{-1/2}$ at 40 GHz	-
Fan (2016) [2]	Integrated opto-mechanical shearing	Pulsed SPDC	1554	± 150	-	106 GHz $W^{-1/2}$ at 8.3 GHz	-
Puigibert (2017) [3]	Serrodyne modulation with bulk LN modulator	Narrow-band SPDC	795	± 20	-	Non-sinusoidal modulation	-
Chen (2021) [4]	IQ-modulation with bulk LN modulator	Narrow-band SPDC	1560	15.65	-	55 GHz $W^{-1/2}$ at 15.65 GHz	-

Karpiński (2017) [5]	EO time lens with bulk LN modulator	Pulsed SPDC	830	-	6.1×	-	2 W at 10 GHz
Mittal (2017) [6]	EO time lens with bulk LN modulator	Pulsed SPDC	1550	-	8.3	-	1.5 W at 3.5 GHz
Sośnicki (2020) [7]	EO time lens with bulk LN modulator	Pulsed SPDC	1560	-	13.7 - 18.4×	-	Non-sinusoidal modulation
**This work**	Integrated TFLN EO modulation	Pulsed SPDC	1560	±641	18.7×	321 GHz $W^{-1/2}$ at 27.5 GHz	0.85 W at 13.6 GHz

2. Modulator design, characterization, and analysis

Modulator RF response analysis. The TFLN waveguide in the modulator has a group velocity of 0.013 cm/ps and a dispersion of -2.3 fs/nm/cm around 1560 nm. The total length of the waveguide, including both modulation region and routing waveguides, is ~7.3 cm, resulting in a group delay dispersion of -16.8 fs/nm. For the largest-bandwidth photon used in the experiments (6.5 nm), assuming transform-limited pulse, this dispersion effect will only broaden the pulse duration from 550.7 fs to 560.7 fs. Consider microwave modulation, our TFLN modulator is designed to be a traveling-wave modulator, where the optical group velocity in the waveguide matches the microwave phase velocity in the transmission line electrode. In the single-pass case, the optical and microwave signals will always be in phase as they travel through the modulator. However, in the double-pass case, there is a section where the optical wavepacket loops back to catch a different microwave cycle (see Fig. S1a). When the wavepacket re-enters the modulator electrode, effective modulation only happens if it sees the same microwave modulation phase as it first enters. The electrode has a length of 2 cm, and the loop-back section has a length of ~2.5 cm, giving a total round trip of 4.5 cm and round-trip delay of 346 ps. We therefore expect the modulator V_π to reach minimum every $\Delta f = 1/346$ ps = 2.89 GHz, matching closely with our measurement results (Fig. S1b). Note that because of the push-pull configuration, the two passes experience modulations with opposite signs. As a result, V_π minimums happen at $(n + 0.5)\Delta f$ instead $n\Delta f$, where $n = 1, 2, 3 \ldots$ Despite the "non-flat" frequency response, spectral shearing and time lensing only requires single-frequency operation. Once the RF frequency is chosen, the shearing and bandwidth compression can be controlled by adjusting the RF power. Therefore, the oscillating V_π does not affect our experiments.

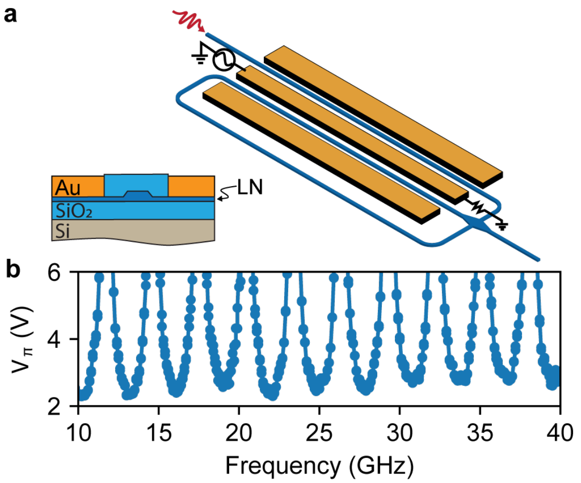

Figure S1. Device geometry and performance. a, Device layout and cross-section of the TFLN electro-optic phase modulator. The modulator adopts a double-pass configuration. This special design increases the interaction length between optical and RF fields, resulting in a reduced V_π. The physical size of the modulator (active area) is 2 cm × 600 µm. **b,** Measured V_π at 1550 nm wavelength**.** Efficient modulation happens when light is in-phase with the RF drive when it re-enters the coplanar waveguide electrode. At these phase-matched frequencies, the measured $V\pi$ is between 2.3-2.8 V from 10 to 40 GHz.

Advantage over single-pass modulator. In a traveling-wave modulator, the RF V_π cannot keep reducing by simply increasing the length. At high frequencies, the RF loss is significant (typically 3 - 4 dB/cm at ~30 GHz). With increasing length, the RF field will soon be fully dissipated and stop contributing to modulation. Moreover, longer modulators will suffer more from velocity mismatch, which may further reduce EO bandwidth. This effect is illustrated in Fig. S2, where we simulated how V_π changes as a function of modulator length using realistic parameters extracted from our TFLN devices. It can be seen that V_π eventually reaches a plateau (dashed line Fig. S2). The double-pass design effectively addresses this problem by passing the waveguide through the electrode twice instead of doubling the length of the electrode. By doing so, with the 2 cm modulator used in our experiment, we already achieved a V_π that a similar single-pass modulator can never achieve. We note that this is something unique about TFLN modulators—the tight mode confinement enables a small bending radius, allowing the waveguide to route back in a compact manner.

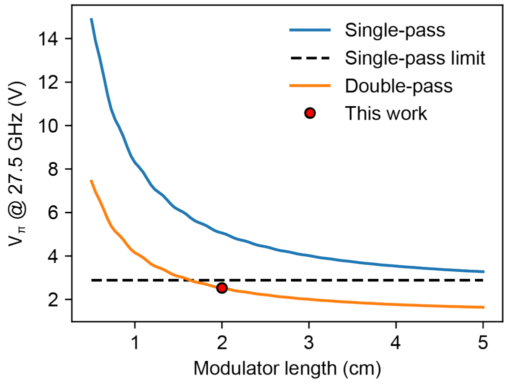

Figure S2. RF V_π comparison between single- and double-pass modulators. The single-pass V_π will eventually plateau due to RF loss, and our device used in this work surpassed this limit. The simulation is performed using parameters from the device used in experiment. Here, RF loss is 0.7 dB $GHz^{-1/2}$ cm^{-1}, optical group index is 2.27, measured RF phase velocity is 2.27, single-pass DC $V_\pi \cdot L$ is 5.8 V-cm, and transmission line impedance is 37 Ω. For the double-pass case, we assumed the modulations are in phase between two passes at this frequency.

Figure S3 compares the EO response among three different modulator designs: (1) 2 cm double-pass modulator used in our experiment, (2) 4 cm single-pass modulator, and (3) 4 cm single-pass modulator with slight velocity mismatch. The low V_π and wide bandwidth of our modulator benefit from careful design of the electrode, where nearly perfect velocity matching was achieved.

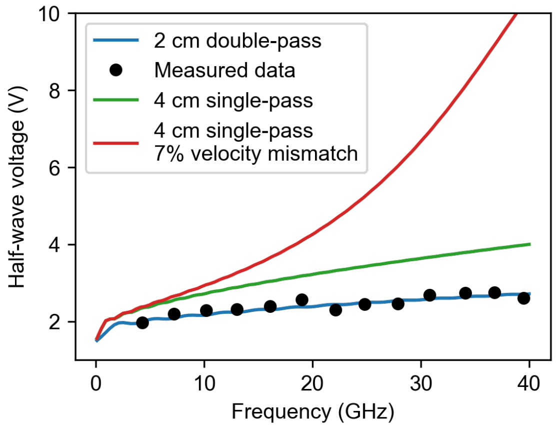

Figure S3. Comparison of RF V_π among different modulator designs. A 2 cm double-pass modulator has lower V_π than a 4 cm single-pass modulator due to smaller RF loss. A slight microwave-optical index mismatch will cause significant increase in V_π at high frequencies. Index mismatch is very common in commercial bulk modulators.

Modulator optical bandwidth. In the main text, we have discussed that ultrafast optical pulses experience negligible dispersion and broadening by passing through the modulator. Here, we further verify that the modulator has large enough optical bandwidth to work with pulsed light. The modulator's optical bandwidth mainly depends on the optical group velocity, which affects velocity matching (determines V_π value) and round-trip time (determines the position of the optimal RF frequency). According to numerical simulation, the group velocity of our TFLN optical waveguide changes by about 0.1% over a bandwidth of 50 nm (much larger than that used in our experiment), causing negligible differences in velocity mismatch. In the loopback waveguide (4.5 cm long), this causes a round trip time difference of about 0.35 ps, which is much smaller than the RF period (~ 36ps for 27.5 GHz). Therefore, we can conclude that the double-pass modulator does not cause problems for pulsed operation in our experiments.

3. Experimental details on spectral shearing, Hong-Ou-Mandel interference, and time lens

Modulation depth measurement. When performing spectral shearing and time lens bandwidth compression, it was difficult to directly measure the RF power applied to the modulator due to the bandwidth limit of our RF spectrum analyzer, the uncalibrated output power of our microwave synthesizer, and various microwave losses from the cables, probes, attenuators, etc. Instead, we estimated the modulation depth (i.e., the number of V_π's) by sending laser light through the modulator and fitting the optical sidebands (measured using a optical spectrum analyzer) with a Bessel function (Fig. S4). Since the V_π values have been independently characterized in Fig. S1b, we can then infer the microwave power applied on the modulator.

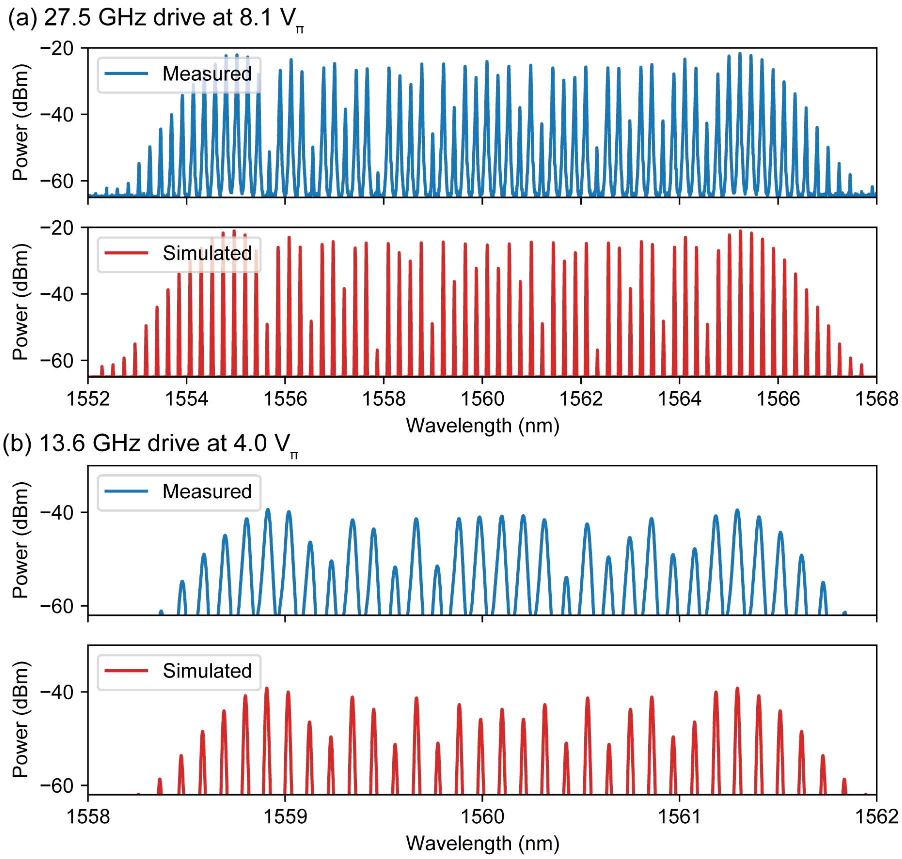

Figure S4. Modulation sidebands when the TFLN modulator was driven at conditions used in the main text. A classical continuous-wave laser was injected into the modulator and the output was measured using an optical spectrum analyzer. The driving amplitude in terms of number of V_π was estimated by fitting the measured spectra using a Bessel function. a, Driving condition in Figure 3 in the main text. b, Driving condition in Figure 4 and 5 in the main text.

Thermal management. During the experiment, there is a significant amount of heat generated on the chip due to RF absorption. The heating primarily causes fiber-to-chip coupling drifts (chip and fiber are mounted on separate stages) rather than affecting the electro-optic performance. The estimated V_π (inferred from frequency shift) has no appreciable difference from that measured at low drive voltages (i.e., no heating). To mitigate heating effect, we glued the chip to a metal sample holder with silver paste and used copper braids to connect the chip holder directly to a thermal sink. During the experiment, we usually turn on the modulation and wait for 10-20 mins to let the system stabilize before performing measurements. This thermal-induced coupling drift can be solved by, for example, directly gluing the fiber to the chip. We repeated the measurements on the same chip over a period of more than 3 months, and each measurement often continued for more than 8 hours. Throughout this period, we did not observe any changes in the EO modulation efficiency or overall optical transmission. We verified the modulator V_π around 4 months after and did not observe any degradation either. In fact, the good thermal stability is somewhat expected since the Curie temperature of LN is > 1000 °C, and the debonding of thin-film lithium niobate is > 500 °C.

HOM interference baseline. We conducted a baseline measurement for the HOM interference. Keeping all other measurement conditions the same (Fig. 4), we set the two spectral filters to have the same center frequencies and performed HOM interference (without applying modulation). We measured interference visibility to be 86.2 ± 3.2% with background subtraction and 73.3 ± 2.8% without background subtraction (see Fig. S5). This proves that the maximum visibility measured in Fig. 4 was likely limited by the measurement setup instead of the shearing process itself.

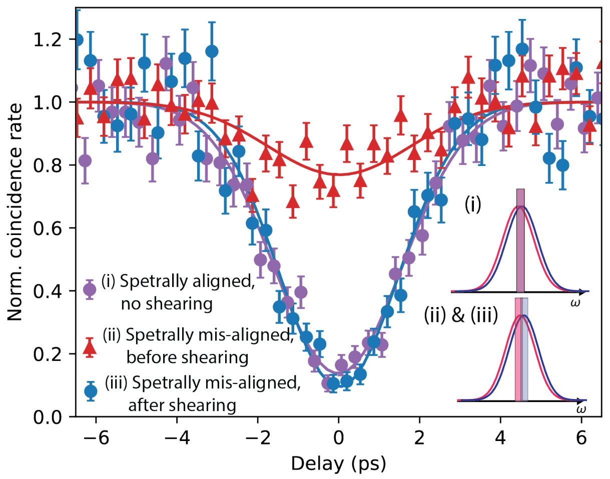

Figure S5. Hong-Ou-Mandel interference before and after spectral shearing. (i) As a starting reference, we aligned the two spectral filters for the two input photons, making them indistinguishable in frequency, and measured interference visibility of 86.2 ± 3.2%. (ii) We then imposed a frequency offset (154 GHz) on the two filters (each with 200 GHz window), making the two input photons different in frequency. This gave a visibility of 23.1 ± 3.1%. (iii) By applying frequency shearing, we frequency shifted one photon and erased the frequency distinguishability, measuring a visibility of 90.5 ± 4.3%. Compared to the reference measurement, we can confirm that the the non-unity visibility after the shearing is not caused by the shearing process, but likely by the measurement setup (such as the resolution and accuracy of the spectral filters, nonoptimal spectral shape of the source, etc.). In the lower right inset, the curves represent the spectral shape of the signal and idler photons from the SPDC source, and the bands represent the spectral filter applied.

Choice of RF frequencies. Given the same RF power, higher RF frequency can result in larger spectral shearing. Our choice of 27.5 GHz is limited by the bandwidth of the available high-power amplifier, which covers 26.2 GHz to 34 GHz. Within this frequency band, 27.5 GHz works best since the amplifier has the highest saturation power while the modulator has the lowest V_π. For the time lens and HOM interference experiment, we intentionally reduced the operating frequency to 13.6 GHz to reduce the timing jitter between the laser pulses and RF drive. The phase-locked oscillator used to drive the modulator is essentially a frequency multiplier (with the laser sync signal as the base frequency), where the frequency noise cascades more with a higher multiplication factor. In our experiment, the timing/frequency jitter started to induce spectral broadening in the shearing process, which reduces the HOM interference visibility and time lens compression factor. In addition, for the time lens experiment, higher RF frequency gives a smaller "temporal aperture." The dispersed optical pulse must be smaller than the RF period to avoid overfilling the aperture and causing aberrations. At higher frequencies, the margin for the dispersion control and time synchronization of the input optical pulse is more stringent.

4. Simulation of bandwidth compression through time lens

In the main text, we have approximated the valley of the sinusoidal phase modulation as a quadratic function, $\phi(t) = Bt^2/2$, with $B = 4\pi^3 \frac{V}{V_\pi} f_{\mathrm{RF}}^2$. This approximation is inaccurate when the input optical pulse has a width comparable to the RF drive period. We therefore numerically simulate the time lens operation without the quadratic function approximation to observe distortion and spurious sidebands when the input pulse overfill the "aperture" of the time lens (Fig. S6).

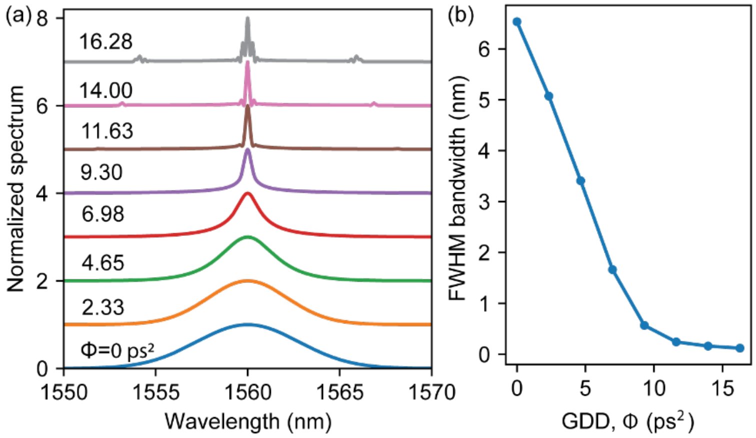

Figure S6. Time lens simulation. Simulated spectral shape (a) and FWHM bandwidth (b) after time lensing with increasing group delay dispersion (GDD) applied. When GDD exceeds ~10 ps^2, the dispersed optical pulse that enters the time lens become too wide and starts to overfill the time lens' effective aperture. As a result, spectral distortion and sidebands are generated. However, these fine features are difficult to resolve in experiments due to limited measurement resolution. Instead, they manifest as an effectively broadened spectrum, as shown in Fig. 5c in the main text. In this simulation, the input photon has a center wavelength of 1560 nm and FWHM bandwidth of 6.55 nm. RF modulation is at 13.6 GHz and 4.0 V_π.

REFERENCES

[1] Wright, L. J. *et al*. Spectral shearing of quantum light pulses by electro-optic phase modulation. *Physical Review Letters* **118**, 023601 (2017).

[2] Fan, L. R. *et al*. Integrated optomechanical single-photon frequency shifter. *Nature Photonics* **10**, 766-770 (2016).

[3] Puigibert, M. G. *et al*. Heralded single photons based on spectral multiplexing and feed-forward control. *Physical Review Letters* **119**, 083601 (2017).

[4] Chen, C. C. *et al*. Single-photon frequency shifting with a quadrature phase-shift keying modulator. *Scientific Reports* **11**, 300 (2021).

[5] Karpiński, M. *et al*. Control and measurement of quantum light pulses for quantum information science and technology. *Advanced Quantum Technologies* **4**, 2000150 (2021).

[6] Mittal, S. *et al*. Temporal and spectral manipulations of correlated photons using a time lens. *Physical Review A* **96**, 043807 (2017).

[7] Sośnicki, F. *et al*. Aperiodic electro-optic time lens for spectral manipulation of single-photon pulses. *Applied Physics Letters* **116**, 234003 (2020).
